# Enhancement of Ovarian Malignancy on Clinical Contrast Enhanced MRI Studies

**DOI:** 10.1155/2013/979345

**Published:** 2013-02-13

**Authors:** Harpreet K. Pannu, Weining Ma, Emily Craig Zabor, Chaya S. Moskowitz, Richard R. Barakat, Hedvig Hricak

**Affiliations:** ^1^Department of Radiology, Memorial Sloan-Kettering Cancer Center, New York, NY 10065, USA; ^2^Department of Epidemiology and Biostatistics, Memorial Sloan-Kettering Cancer Center, New York, NY 10065, USA; ^3^Gynecology Service, Department of Surgery, Memorial Sloan-Kettering Cancer Center, New York, NY 10065, USA

## Abstract

*Purpose*. To assess if there is a significant difference in enhancement of high grade serous carcinoma of the ovary compared with other ovarian malignancies on clinically performed contrast enhanced MRI studies. *Methods*. In this institutional-review–board-approved study, two radiologists reviewed contrast enhanced MRI scans in 37 patients with ovarian cancer. Readers measured the signal intensity (SI) of ovarian mass and gluteal fat pre- and postcontrast administration. Percentage enhancement (PE) was calculated as [(post-pre)/precontrast SI] × 100. *Results*. Pathology revealed 19 patients with unilateral and 18 patients with bilateral malignancies for a total of 55 malignant ovaries-high grade serous carcinoma in 25/55 ovaries (45%), other epithelial carcinomas in 12 ovaries (22%), nonepithelial cancers in 8 ovaries (14%), and borderline tumors in 10 ovaries (18%). Enhancement of high grade serous carcinoma was not significantly different from other invasive ovarian malignancies (Reader 1 *P* = 0.865; Reader 2 *P* = 0.353). Enhancement of invasive ovarian malignancies was more than borderline tumors but did not reach statistical significance (Reader 1*P* = 0.102; Reader 2 *P* = 0.072). *Conclusion*. On clinically performed contrast enhanced MRI studies, enhancement of high grade serous ovarian carcinoma is not significantly different from other ovarian malignancies.

## 1. Introduction

Epithelial ovarian carcinoma is no longer felt to be a uniform disease, both in the pathology and oncology literature. It is now divided into two categories in pathology articles, tumors arising from a precursor lesion with a better prognosis versus tumors arising de novo with a worse prognosis [1]. The most common type of epithelial ovarian carcinoma, high grade serous carcinoma, is in the second category [2]. These two categories are based on differences in genetic mutations with clinical implications for targeted chemotherapy [1, 3]. Cytotoxic and antiangiogenesis medications targeting various cellular receptors and pathways have the potential to improve response rates in patients [3]. Angiogenesis or tumor vascularity can be indirectly assessed on imaging by the degree of enhancement of the mass.

Although ovarian carcinomas are now viewed as two distinct groups in the pathology literature, little is known about whether there are imaging differences between the groups. Thus far, most imaging studies of ovarian tumors, including those using dynamic contrast enhanced (DCE) MRI, have not evaluated the enhancement of the subtypes of ovarian cancer and only a minority of patients in these reports have had serous carcinoma [4–10]. For ovarian masses, the focus in imaging has been on differentiating benign from malignant masses [4–10]. In contrast, on imaging studies for tumors such as renal cell carcinoma, it is recognized that the histologic subtypes have different enhancement characteristics, for example, papillary versus clear cell, and this difference is evident on postcontrast scans obtained in the clinical setting [11–13]. However, it is not known if similar observations can be made for the subtypes of ovarian cancer. Therefore, we performed a pilot study to determine if there is a significant difference in enhancement to distinguish high grade serous carcinoma from other ovarian cancers in routine clinical practice.

## 2. Materials and Methods

### 2.1. Patient Population

The Institutional Review Board issued a waiver of informed consent for our retrospective study, which was conducted in compliance with HIPAA guidelines. The pathology database was searched to identify patients who had ovarian cancer at oophorectomy between December 2004 and January 2011, followed by a search for preoperative MRI studies in this group. Inclusion criterion was the availability of MR scans with pre- and postcontrast gradient echo MR imaging of the pelvis with fat saturation, and 37 scans in 37 patients met this criterion and formed the study group. The median age of the patients was 59 years (range 16–83 years). The median time interval between MRI and oophorectomy was 21 days (mean 25 days, range 6–65 days). The MRI was done prior to chemotherapy or any other treatment in all patients with primary ovarian cancer in our study.

Pathology reports were reviewed following analysis of the MRI scans. For data analysis, patients were divided into 4 categories based on histology of the malignant ovary: high grade serous carcinoma, other epithelial ovarian carcinoma, other nonepithelial ovarian cancer, and borderline tumor.

### 2.2. Imaging Technique

All MRI scans were performed with fat saturated gradient echo (GRE) sequences before and after intravenous gadolinium contrast administration; 3D-GRE technique was used in 28 patients and 2D-GRE technique was used in 9 patients. The scans were performed between 2004 and January 2011 at multiple institutions on magnets from different vendors and were retrospectively reviewed at our institution on a single picture archiving and communication system (PACS) (Centricity, GE Medical Systems, Milwaukee, WI, USA). 3D-GRE scan parameters were as follows: median TR 4.4 ms (range 3–9.1 ms), median TE 1.9 ms (range 0.85–2.9 ms), median slice thickness 2.5 mm (range 1.5–9 mm), median NEX 0.75 (range 0.37–1), median field of view 26 × 24 cm (range 20 × 38 cm), median frequency matrix 256 (range 156–320), and median phase matrix 160 (range 98–256). 2D-GRE scan parameters were as follows: median TR 250 ms (range 172–520 ms), median TE 3.7 ms (range 1.2–5.6 ms), median slice thickness 5.5 mm (range 4.5-8.5 mm), NEX 1, median field of view 28 × 24 cm (range 20 × 34 cm), median frequency matrix 256 (range 232–320), and median phase matrix 166 (range 128–224). Four postcontrast phases were performed in 12 patients, 3 phases in 17 patients, 2 phases in 3 patients, and 1 phase in 5 patients. The first postcontrast scan was obtained in the axial plane in 32 patients, in the sagittal plane in 3 patients, and in the coronal plane in 2 patients. The planes of the first and second postcontrast scans differed in 14 patients.

### 2.3. Scan Analysis

The MRI scans were independently reviewed by two readers, each with several years of experience in gynecologic imaging. The readers were blinded to the pathology of the ovarian mass. Each reader noted the presence and size or the absence of any ovarian mass. The highest enhancing solid component was selected for signal intensity (SI) measurement with the largest possible circular region of interest (ROI). This ROI was placed on the same region of enhancing tissue on all postcontrast phases, and cross-referencing was performed when necessary. Mean ROI size was 43.1 mm^2^ (range 1.8–177.5 mm^2^) for Reader 1 and 56.8 mm^2^ (range 7–291 mm^2^) for Reader 2.

Percent enhancement of the lesion was calculated as [(SI lesion post − SI lesion pre)/SI lesion pre] × 100 where SI post = lesion signal intensity on post contrast scan and SI pre = lesion signal intensity on pre contrast scan. In addition, to compare the SI of other pelvic structures between the pre and postcontrast scans, the SI of the buttock fat immediately superficial to the gluteus muscle was obtained for all phases. The ratio of the lesion SI to fat SI was calculated for the same phase in each patient and percentage of change in the lesion/fat SI ratio from pre- to post contrast.

Since the delay between contrast injection and scan acquisition was not available from retrospective review, SI of the right external iliac artery and vein was also obtained for each phase at the level of the ovarian mass to calculate an arterial/venous ratio.

### 2.4. Statistical Analysis

All patients had a precontrast enhancement measurement and between 1 and 4 post-contrast enhancement measurements. The percentage change in enhancement between the pre-contrast measurement and each post-contrast measurement was calculated, and the maximum of these values was taken to be the highest percent enhancement (HPE). Percentage change in enhancement between consecutive pairs of phase was also calculated. Enhancement was summarized using the median and range of the values across participants. HPE was also summarized by pathologic type of primary tumor. The change in lesion/fat ratio between the pre-contrast measurement and each post-contrast measurement was also calculated and the maximum was found. The arterial to venous (A/V) ratio was calculated at each phase and the maximum was found. We examined the association between maximum A/V ratio and the highest percent enhancement using scatterplots. Finally, comparisons of HPE and maximum change in lesion/fat ratio were calculated using generalized estimating equations (GEE) models with an exchangeable correlation structure. GEE models employ a sandwich variance estimate to account for the correlation between multiple ovarian enhancement measures from a single patient. Lesion and lesion/fat HPE were transformed to the log scale for modeling purposes. Interreader agreement was calculated using the concordance correlation coefficient (CCC). Results of all analyses are presented separately for the two independent readers and *P* values < 0.05 were considered statistically significant. All analyses were performed using SAS software, version 9.2 for Windows (SAS Institute, Cary, NC, USA) and R version 2.11.0 (R Development Core Team; 2010).

## 3. Results

### 3.1. Pathologic Findings

Pathology revealed a total of 55 malignant ovaries in the study population of 37 patients, and 18 patients had bilateral malignancy and 19 patients had unilateral malignancy. High grade serous carcinoma occurred in 25 ovaries (46%), other epithelial carcinomas (well or moderately differentiated endometrioid carcinoma, clear cell carcinoma, mucinous carcinoma, and carcinosarcoma) occurred in 12 ovaries (22%), other non-epithelial cancers (carcinoid, dysgerminoma, immature teratoma, struma ovarii, granulosa cell tumor, and metastatic breast cancer) occurred in 8 ovaries (14%), and borderline tumors occurred in 10 ovaries (18%).

In the 18 patients with bilateral malignant ovaries, the same histology was seen bilaterally in 12 patients with high grade serous carcinoma, 2 patients with borderline tumor, 1 patient with carcinosarcoma, and in 1 patient with metastatic breast cancer. Two patients had an endometrioid carcinoma in one ovary and a borderline tumor in the other ovary.

Staging in the patients with primary ovarian cancer was as follows. Sixteen patients had stage I disease (43%), 1 patient had stage II disease (3%), 15 patients had stage III disease (41%), and 2 patients had stage IV disease (5%). Precise staging was not available for the remaining patients (*n* = 3, 8%), 1 of whom was clinically classified as having advanced disease. Thirty of the 37 patients (81%) had optimal surgical debulking defined as residual disease <1 cm.

### 3.2. Imaging Findings

The average size of the largest ovarian mass in each patient was 9.3 ± 5.1 cm as measured by Reader 1. Tables 1 and 2 summarize the enhancement of the ovarian masses by histologic type and reader.  Table 1 gives the percentage of lesion enhancement while Table 2 gives the percentage of (lesion/fat) enhancement which accounts for possible changes in technical parameters between pre- and postcontrast scans and therefore has lower enhancement values compared with Table 1.

On the first postcontrast phase, the mean and median percent enhancement of ovarian malignancies was less than 100% in most cases indicating that the signal intensity of the tumor group as a whole did not double. Including all post contrast phases, the highest mean and median percent enhancement seen was between 100% and 200% for the invasive tumors and approaching 100% for borderline tumors.

**Figure 1:**
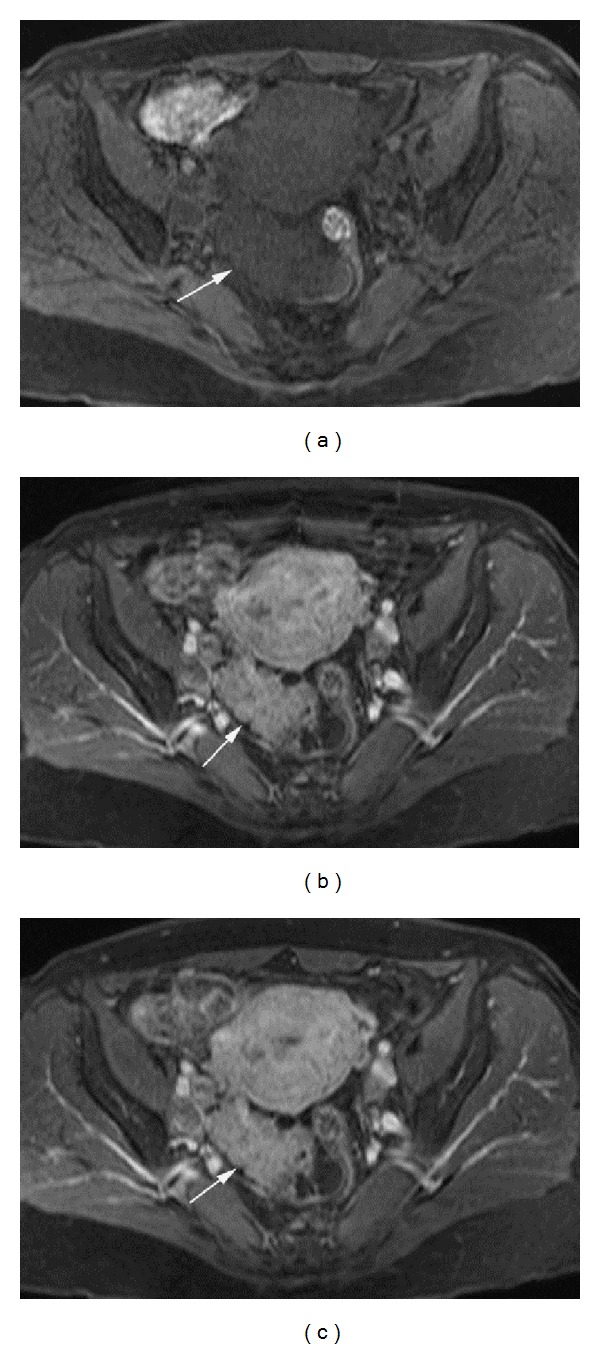
Enhancement of high grade serous carcinoma of the ovary in a postmenopausal patient. Postmenopausal patient in her mid 50s with high grade serous carcinoma of the right ovary. (a) Precontrast, (b) first postcontrast phase, and (c) third postcontrast phase axial 3D gradient echo T1 weighted images of the pelvis show enhancement of the solid component (arrow) of a right adnexal mass. Percentage of lesion enhancement on the first postcontrast phase was 272.6% for Reader 1 and 275.1% for Reader 2. Percent (lesion/fat) enhancement on the first postcontrast phase was 218.9% for Reader 1 and 171.3% for Reader 2.

**Figure 2:**
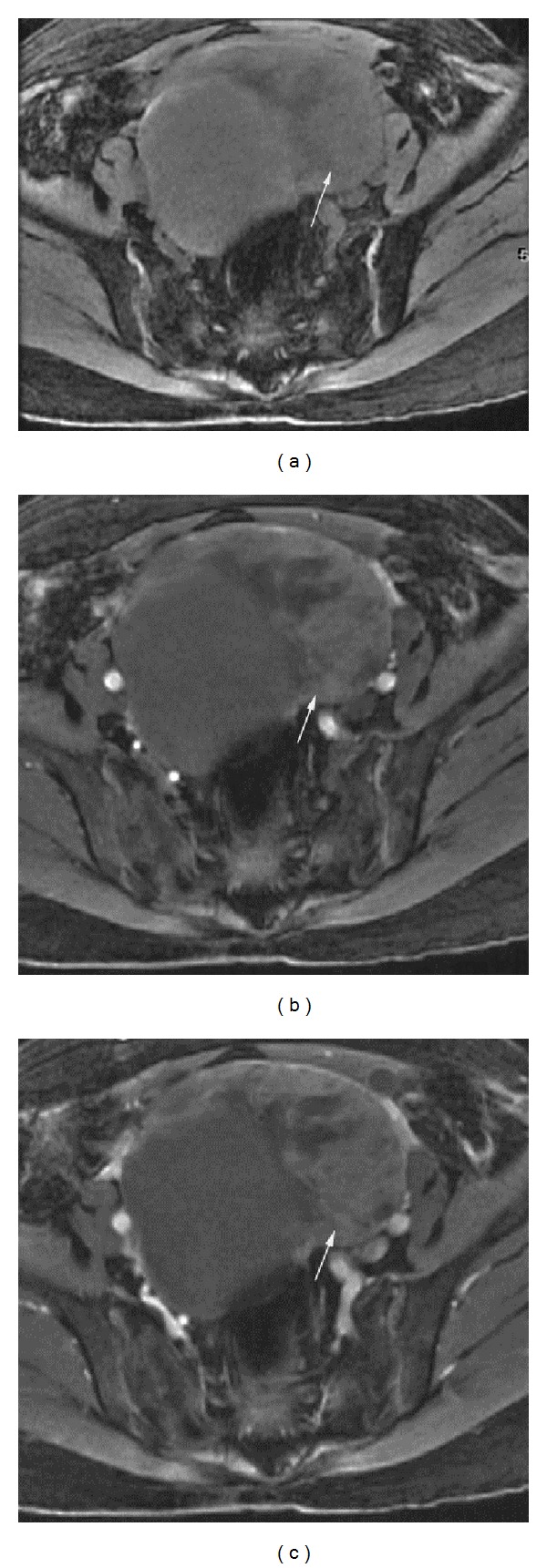
Enhancement of high grade serous carcinoma of the ovary in a postmenopausal patient. Postmenopausal patient in her mid 60s with high grade serous carcinoma of the left ovary. (a) Pre contrast, (b) first post contrast phase, and (c) third post contrast phase axial 3D gradient echo T1 weighted images of the pelvis show mild enhancement of the solid component of large pelvic mass (arrow) in left side of pelvis. Cystic component of mass is on right side of pelvis. Percentage of lesion enhancement on the first post contrast phase was 74.0% for Reader 1 and 22.1% for Reader 2. Percent (lesion/fat) enhancement on the first post contrast phase was 58.3% for Reader 1 and 20.7% for Reader 2.

**Figure 3:**
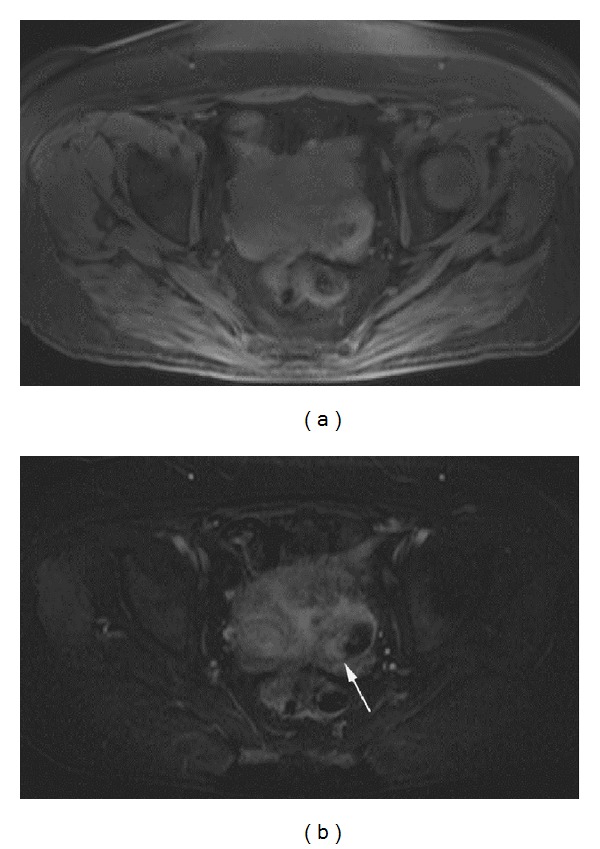
Enhancement of endometrioid carcinoma of the ovary in a postmenopausal patient. Postmenopausal patient in her early 60 s with moderately differentiated endometrioid carcinoma of the left ovary. (a) Precontrast and (b) subtraction image of first post contrast phase axial 3D gradient echo T1 weighted images of the pelvis show a heterogeneous left pelvic mass with enhancement of the nodular soft tissue component (arrow). The mass had a proteinaceous cystic component and also invaded the uterus. Percentage of lesion enhancement on the first post contrast phase was 156.8% for Reader 1 and 117.7% for Reader 2. Percent (lesion/fat) enhancement on the first post contrast phase was 126.9% for Reader 1 and 88.4% for Reader 2.

**Figure 4:**
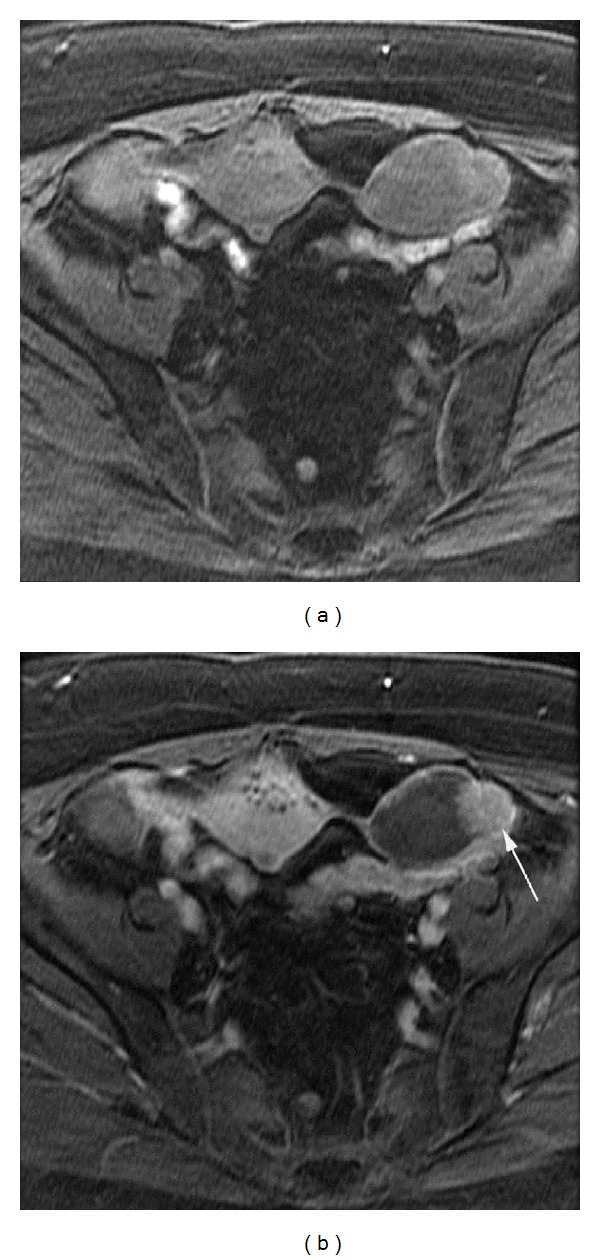
Enhancement of borderline tumor of the ovary in a postmenopausal patient. Postmenopausal patient in her late 50s with serous borderline tumor of the left ovary. (a) Precontrast and (b) third postcontrast phase axial 3D gradient echo T1 weighted images of the pelvis show enhancement of the nodular soft tissue component of the mass (arrow). Percentage of lesion enhancement on the first post contrast phase was 72.9% for Reader 1 and 66.3% for Reader 2. Percent (lesion/fat) enhancement on the first post contrast phase was 18.6% for Reader 1 and 17.2% for Reader 2.

Table 3 shows the comparison of enhancement of the different tumor groups. The percent enhancement of high grade serous carcinoma was not significantly different from other invasive ovarian malignancies (Reader 1 *P* = 0.865; Reader 2 *P* = 0.353). The percent enhancement of invasive ovarian malignancies was more than borderline tumors and approached statistical significance (Reader 1 *P* = 0.102; Reader 2 *P* = 0.072). Illustrative examples of the enhancement of high grade serous carcinoma, endometrioid carcinoma, and borderline tumor are shown in Figures 1, 2, 3, and 4.

**Figure 5:**
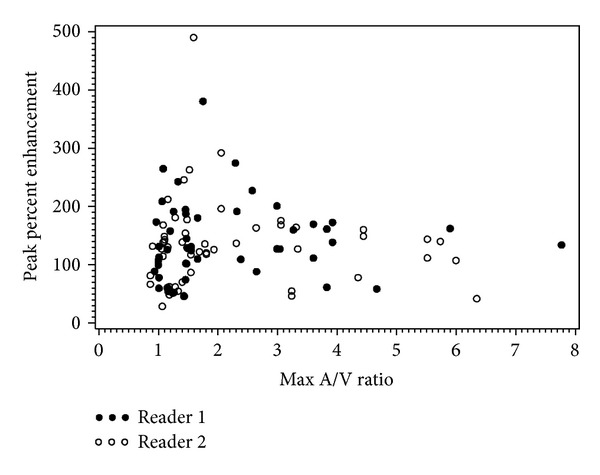
*Y* axis: highest lesion percentage of enhancement seen. Enhancement = [(postcontrast lesion SI − precontrast lesion SI)/precontrast lesion SI] × 100. *X* axis: highest ratio (external iliac artery SI/external iliac vein SI) seen on post contrast phases.

There was variability in the enhancement of all tumor groups as seen in the wide range of enhancement values in Tables 1 and 2 and Figures 1 and 2. The spread of enhancement values versus the calculated arterial/venous ratio is plotted in Figure 5. Higher degrees of enhancement were not seen with higher arterial/venous ratios. The mean peak arterial/venous SI ratio for the 3D MRI scans (*n* = 28 scans) was 2.2 (median 1.5, range 0.8–7.7) and for the 2D MRI scans (*n* = 9 scans) was 1.3 (median 1, range 0.7–3.0). The concordance correlation coefficient (95% CI) for agreement between the two readers on peak percent enhancement of the lesion was 0.80 (95% CI: 0.70, 0.90).

## 4. Discussion

In our study of clinically performed MRI scans, there was no significant difference in the enhancement of high grade serous carcinoma compared to other invasive ovarian cancers. These results suggest that in clinical practice enhancement cannot be used to distinguish high grade serous carcinoma from other ovarian malignancies. In addition, there was also a wide range in the degree of enhancement of all the tumor groups including high grade serous carcinoma as shown in Table 1 and in Figures 1 and 2. Although some of this variability is possibly due to technical factors such as contrast injection and scan delay, a plot of enhancement versus arterial/venous ratio in Figure 5 did not show a pattern for higher enhancement with higher arterial/venous ratios suggesting that other factors may also play a role.

Enhancement of ovarian masses depends on the delivery and retention of contrast in the lesion. The vascular supply, capillary network, and leakage of contrast into the extravascular interstitial space contribute to the accumulation of contrast within the mass and greater enhancement [14]. Angiogenesis is known to occur in cancers and supports tumor growth [14, 15]. However, microvascular density alone may not correlate with the degree of enhancement and other factors are also likely responsible [7]. The pericyte coverage index which is a measure of vascular maturity has been reported to correlate negatively with enhancement amplitude [7]. Vascular endothelial growth factor receptor (VEGFR-2) which affects angiogenesis and vascular permeability has also been shown to be expressed by more cells in invasive tumors compared to benign masses [7]. However, variable genetic aberrations may result in variable expression of VEGFR-2 as well as other factors [16]. These genetic variations may occur within a histologic subtype. An analysis of genetic mutations in high grade serous carcinoma found TP53 mutations in the majority of cases (96%) but a lower prevalence of mutations in other genes [17]. Using the renal cell carcinoma model, a recent study on clear cell renal cell carcinoma showed that tumors with different chromosomal aberrations showed different levels of enhancement on CT [18]. This suggests that the morphologic appearance can differ even within the same histologic subtype of tumor depending on the genetic makeup. In addition, these genetic variations could account for overlap in the morphologic appearance of the different histologic subtypes of ovarian cancer [16]. More detailed analysis correlating genetic analysis with imaging would shed light on the enhancement of ovarian malignancies.

Compared to a DCE-MRI study by Thomassin-Naggara et al. which focused on distinguishing malignant from benign ovarian lesions, there was lower mean percent enhancement of the ovarian malignancies in our study [7]. This is expected given the probable higher contrast injection rate, smaller scan delay, and higher temporal resolution with DCE-MRI. In their study, a preselected solid portion of the tumor was imaged at 5-second intervals following injection of contrast at 2 cc/second [7]. The study analyzed ovarian masses from 41 patients, 16 of whom had invasive epithelial ovarian carcinomas, and the median percent enhancement of the invasive masses was 176.6% (range 129.1–225.5%) [7]. The majority of tumors in this study by Thomassin-Naggara et al. were endometrioid carcinomas compared to high grade serous carcinoma in our study.

The publications by Dilks et al. have imaged the entire ovarian mass at 30-second intervals after contrast and selected the highest enhancing component to measure signal intensity similar to the image review method used in our study [9, 10]. An initial publication by this group demonstrated higher percentage enhancement and wash-in rate in ovarian malignancies compared with benign masses [9]. However, the malignant group had only one case of high grade serous carcinoma and included borderline tumors limiting conclusions about the different histologic subtypes of ovarian cancer [9]. A more recent publication by the same group included 8 cases of serous cystadenocarcinoma as well as borderline tumors in a cohort of 36 malignant lesions [10]. The patients were imaged at 30-second intervals for 2 minutes and malignant tumors showed a percentage enhancement of 81 ± 33.5% [10]. This is similar to the degree of enhancement seen in our study on the first post contrast phase.

When the authors separated borderline from invasive tumors, malignant tumors had a percentage enhancement of 89.5 ± 28.8% compared with 38.8 ± 22.1% for borderline tumors and this difference was statistically significant (*P* = 0.001) [10]. In the study by Thomassin-Naggara et al., the median percent enhancement of invasive tumors was also higher than borderline tumors measuring 176.6% (range 129.1–225.5%) versus 79.3% (range 55.5–155.3%) [7]. Similar to both these prior DCE MRI studies, invasive ovarian tumors in our clinical study showed higher enhancement compared with borderline ones [7, 10]. This may be due to variable angiogenesis and VEGF expression by borderline tumors [16].

A limitation of our study is that clinically performed studies were reviewed retrospectively and it is possible that the post contrast phases were delayed resulting in an underestimation of the degree of enhancement of the ovarian mass due to washout. Overall, the highest percentage of enhancement achieved (Table 1) was greater than the percentage of enhancement in the first post contrast phase suggesting continued enhancement of the mass on subsequent phases. In addition, previous MRI studies with contrast kinetics time curves suggest that rapid washout of contrast is not typical in the initial imaging time period [5, 7, 8, 19]. One MRI study with various histologic subtypes of ovarian cancer showed overall progressive enhancement of malignant ovarian masses from 60 seconds to 120 seconds [5]. Other limitations of our study include technical differences between the scans, such as the use of both 2D and 3D technique and the lack of a standardized imaging protocol.

## 5. Conclusion

On clinically performed contrast enhanced MRI studies, enhancement of high grade serous ovarian carcinoma is not significantly different from other ovarian malignancies. Invasive tumors enhanced greater than borderline tumors.

Unlike the model of renal cell carcinoma where differing enhancement of papillary and clear cell types is seen both qualitatively and quantitatively in clinical practice without strict adherence to scan timing, such a difference was not evident in our study on the subtypes of ovarian cancer. The role of wash-in rate of contrast may be helpful in learning more about the enhancement of high grade serous carcinoma and is an added value of DCE-MRI scans over clinically performed MRI. High temporal resolution scanning and quantification of contrast kinetics in ovarian cancers may have a potential role in the imaging evaluation of patients [19, 20].

## Figures and Tables

**Table 1:** Lesion enhancement. Enhancement characteristics of lesion stratified by type of ovarian pathology and reader.

Enhancement characteristics of lesion by ovarian pathology and reader.					
Pathology	% lesion enhancement	Reader 1		Reader 2	
		Mean (SD)	Median (range)	Mean (SD)	Median (range)
High grade serous carcinoma	Post 1	83.1 (64.9)	76.1 (11.3, 272.6)	81.0 (64.3)	75.6 (−11.4, 275.1)
	Highest enhancement	136.9 (78.5)	126.7 (54.6, 380.7)	136.8 (95.7)	119.5 (46.8, 490.2)
Other epithelial carcinoma	Post 1	136.0 (62.0)	148.4 (11.0, 227.7)	130.1 (58.9)	133.8 (10.2, 246.1)
	Highest enhancement	163.9 (58.8)	177.1 (52.4, 265.0)	150.9 (53.1)	144.4 (28.9, 246.1)
Other nonepithelial cancer	Post 1	76.9 (78.5)	74.4 (−13.8, 209.0)	66.4 (52.9)	57.2 (−2.2, 144.3)
	Highest enhancement	151.8 (59.1)	160.9 (61.6, 242.7)	163.6 (44.3)	153.4 (122.4, 263.2)
Borderline tumor	Post 1	57.2 (34.4)	66.0 (8.4, 100.9)	58.4 (48.6)	62.3 (−0.6, 155.7)
	Highest enhancement	98.0 (46.4)	102.3 (45.8, 191.7)	93.4 (47.8)	74.8 (42.1, 177.7)

Post 1: enhancement on first postcontrast phase.

Highest: highest enhancement demonstrated on all postcontrast phases.

% lesion enhancement = [(postcontrast lesion SI − precontrast lesion SI)/precontrast lesion SI] × 100.

SI: signal intensity.

**Table 2:** Lesion/fat ratio enhancement. Enhancement characteristics of (lesion/fat) ratio stratified by type of ovarian pathology and reader.

Enhancement characteristics of lesion/fat ratio by ovarian pathology and reader.					
Pathology	% (lesion/fat) enhancement	Reader 1		Reader 2	
		Mean (SD)	Median (range)	Mean (SD)	Median (range)
High grade serous carcinoma	Post 1	77.8 (88.1)	55.5 (2.6, 358.1)	106.4 (147.4)	63.1 (−1.7, 610.8)
	Highest enhancement	160.1 (124.3)	102.1 (7.0, 433.1)	160.4 (142.4)	106.2 (34.7, 610.8)
Other epithelial carcinoma	Post 1	90.1 (63.3)	72.1 (11.3, 202.2)	82.8 (38.2)	76.7 (33.1, 151.3)
	Highest enhancement	138.9 (88.9)	126.6 (23.2, 320.3)	216.4 (184.5)	178.4 (48.6, 698.6)
Other non-epithelial cancer	Post 1	67.4 (87.7)	45.3 (−27.4, 208.3)	83.6 (114.9)	45.0 (−8.1, 332.9)
	Highest enhancement	129.3 (111.3)	90.0 (20.1, 363.9)	148.2 (80.8)	130.5 (63.3, 332.9)
Borderline tumor	Post 1	29.2 (27.5)	35.6 (−22.1, 61.1)	26.8 (23.5)	27.8 (0.0, 71.6)
	Highest enhancement	92.5 (51.9)	82.0 (17.6, 180.3)	138.8 (117.2)	83.8 (0.0, 284.9)

Post 1: enhancement on first post contrast phase.

Highest: highest enhancement demonstrated on all post contrast phases.

Lesion/fat = ratio of lesion SI/fat SI on same phase. SI: signal intensity.

% (Lesion/fat) enhancement = [(postcontrast lesion/fat SI − precontrast lesion/fat SI)/precontrast lesion/fat SI] × 100.

**Table 3:** Table of *P* values comparing enhancement of different pathology groups.

*P* values comparing enhancement of different pathology groups		
	Reader 1	Reader 2
Highest % lesion enhancement		

High grade serous carcinoma versus all other pathologies	0.665	0.981
High grade serous carcinoma versus other invasive pathologies	0.297	0.457
Borderline versus invasive pathologies	0.014	0.040

Highest % (lesion/fat) enhancement		

High grade serous carcinoma versus all other pathologies	0.813	0.716
High grade serous carcinoma versus other invasive pathologies	0.865	0.353
Borderline versus invasive pathologies	0.102	0.072

All other pathologies = other epithelial carcinomas + other non-epithelial cancers + borderline tumors.

Other invasive pathologies = other epithelial carcinomas + other non-epithelial cancers.

Invasive pathologies = high grade serous carcinoma + other epithelial carcinomas + other non-epithelial cancers.

*P* values from GEE models accounting for correlation between multiple ovarian enhancement measures from a single patient using a sandwich variance estimate. An exchangeable correlation structure was used. The log of peak percent lesion enhancement and the log of peak percent lesion/fat ratio enhancement were used in modeling.
